# A variation in *PANK2* gene is causing Pantothenate kinase-associated Neurodegeneration in a family from Jammu and Kashmir – India

**DOI:** 10.1038/s41598-017-05388-9

**Published:** 2017-07-05

**Authors:** Arshia Angural, Inderpal Singh, Ankit Mahajan, Pranav Pandoh, Manoj K Dhar, Sanjana Kaul, Vijeshwar Verma, Ekta Rai, Sushil Razdan, Kamal Kishore Pandita, Swarkar Sharma

**Affiliations:** 1 Human Genetics Research Group, Department of Biotechnology, Shri Mata Vaishno Devi University, Katra, Jammu and Kashmir 182320 India; 2 Bioinformatics Infrastructure Facility, Department of Biotechnology, Shri Mata Vaishno Devi University, Katra, Jammu and Kashmir 182320 India; 3 School of Biotechnology, University of Jammu, Jammu and Kashmir, 180006 India; 4 Acharya Shri Chander College of Medical Sciences and Hospital, Sidra, Jammu, Jammu and Kashmir 180017 India; 5 Neurology Clinic, 7 Bhagwati Nagar, Jammu, 180001 J&K India; 6 Health Clinic, H. No. 62, Lane 11, Swam Vihar, Muthi, Jammu, 181205 J&K India

## Abstract

Pantothenate kinase-associated neurodegeneration is a rare hereditary neurodegenerative disorder associated with nucleotide variation(s) in mitochondrial human Pantothenate kinase 2 (hPanK2) protein encoding *PANK2* gene, and is characterized by symptoms of extra-pyramidal dysfunction and accumulation of non-heme iron predominantly in the basal ganglia of the brain. In this study, we describe a familial case of PKAN from the State of Jammu and Kashmir (J&K), India based on the clinical findings and genetic screening of two affected siblings born to consanguineous normal parents. The patients present with early-onset, progressive extrapyramidal dysfunction, and brain Magnetic Resonance imaging (MRI) suggestive of symmetrical iron deposition in the globus pallidi. Screening the *PANK2* gene in the patients as well as their unaffected family members revealed a functional single nucleotide variation, perfectly segregating in the patient’s family in an autosomal recessive mode of inheritance. We also provide the results of *in-silico* analyses, predicting the functional consequence of the identified *PANK2* variant.

## Introduction

Pantothenate Kinase-Associated Neurodegeneration (PKAN; OMIM# 234200) is a rare autosomal recessive, progressive neurodegenerative movement disorder characterized by accumulation of non-heme iron in the brain, predominantly in the bilateral globus pallidus region of the basal ganglia^1–3^. Also known as Neurodegeneration with Brain Iron Accumulation (NBIA) Type-1 (NBIA-1), it belongs to a spectrum of inherited neurodegenerative disorders, namely NBIA that shares a common detectable feature of iron deposition in basal ganglia on the brain MRI of the patients. Being the most common type of NBIA, PKAN accounts nearly 30–50% of the global NBIA cases with an estimated global prevalence of about 1 to 3 in one million individuals^3, 4^. The age-of-onset of symptoms is from infancy to late adulthood with variable rate of progression and severity of the motor symptoms, based on which the disorder is clinically classified into two phenotypes –a “classic or typical or early-onset PKAN” with a rapid progression and clinically homogenous phenotype in majority of the cases and “atypical or late-onset PKAN” with a slower progression and variable clinical phenotypes^4, 5^. Further, both familial and sporadic cases of PKAN are known^6, 7^.

The diagnosis of PKAN is generally established on patient’s clinical, radiological as well as genetic backgrounds. The main clinical feature includes progressive neurodegeneration due to non-heme iron overload in the globus pallidus and/or the pars reticularis of the substantia nigra. Many of the times, it is observed as an emblematic “eye-of-the-tiger” sign on cranial MRI of the affected individuals, demonstrated as a T2-weighted (T2W) hypointense signal with an antero-medial hyperintensity between externa and interna of the globus pallidus^2–4, 8–12^. However, reports exist that the hypointense afflicted iron rich globus pallidi displaying “eye-of the-tiger” sign gets attenuated or undergoes “blooming effect” on Gradient Echo T2*-weighted (GRE-T2*W) and Susceptibility Weighted Imaging (SWI) sequences^13–15^. Affected individuals predominantly suffer from progressive extra-pyramidal dysfunction, demonstrated by dystonia, rigidity and choreoathetosis as well as optic atrophy and retinal degeneration^3, 9^. The additional progressive clinical manifestations include dysarthria, intellectual impairment, psychiatric problems, spasticity, and others as have been described elsewhere^3, 7, 9, 10, 13, 16^. Autopsy findings of the afflicted brain regions depict areas of non-heme iron accumulation, presence of axonal swellings or spheroids and loss of neurons^17–20^.

PKAN is usually classified as an inborn error of mitochondrial CoA biosynthesis from pantothenic acid associated with nucleotide variation(s) in ubiquitously expressed mitochondrial hPanK2 protein (EC 2.7.1.33) encoding *PANK2* gene (20p13-p12.3; OMIM# 606157) that encodes a 1.85 kb long transcript having 7 exons^1, 2, 5, 21, 22^. hPanK2 catalyzes the first rate-limiting step, that is, ATP-dependent phosphorylation of pantothenic acid to 4′-phosphopantothenate, of the universal biosynthesis pathway of CoA^1^. It has been hypothesized that a defective mitochondrial hPanK2 transcribed by *PANK2* gene having functional variations can lead to CoA deficiency or defective CoA biosynthesis and subsequently to accumulation of iron and toxic secondary metabolites downstream of the CoA biosynthesis pathway, resulting in the characteristic disease phenotype in PKAN-affected individuals^23^. However, the molecular pathogenesis of PKAN is not fully understood yet.

In this article, we report on a familial case of the early-onset classic PKAN from Jammu and Kashmir State (J&K), India, established on the clinical, radiological and genetic backgrounds of two affected siblings, elder 25 years old female (proband) and younger 23 years old male. They were born of a consanguineous marriage at a full-term pregnancy and presented with neurological complaints and a history of progressive movement disorder. No other family members had a similar problem as well as other movement disorder during their lifetime. Through genetic screening of the affected individuals as well as their unaffected family members, a pathogenic single nucleotide missense variation NM_153638.3:c. 1069C > T (p. Arg357Trp) (rs753376100) was found in exon 3 of *PANK2* gene. Here, we also provide results of the *in-silico* analyses predicting the probable functional consequences of the found *PANK2* variation on the overall dynamics of hPanK2 protein.

## Case Presentation

**Figure 1:**
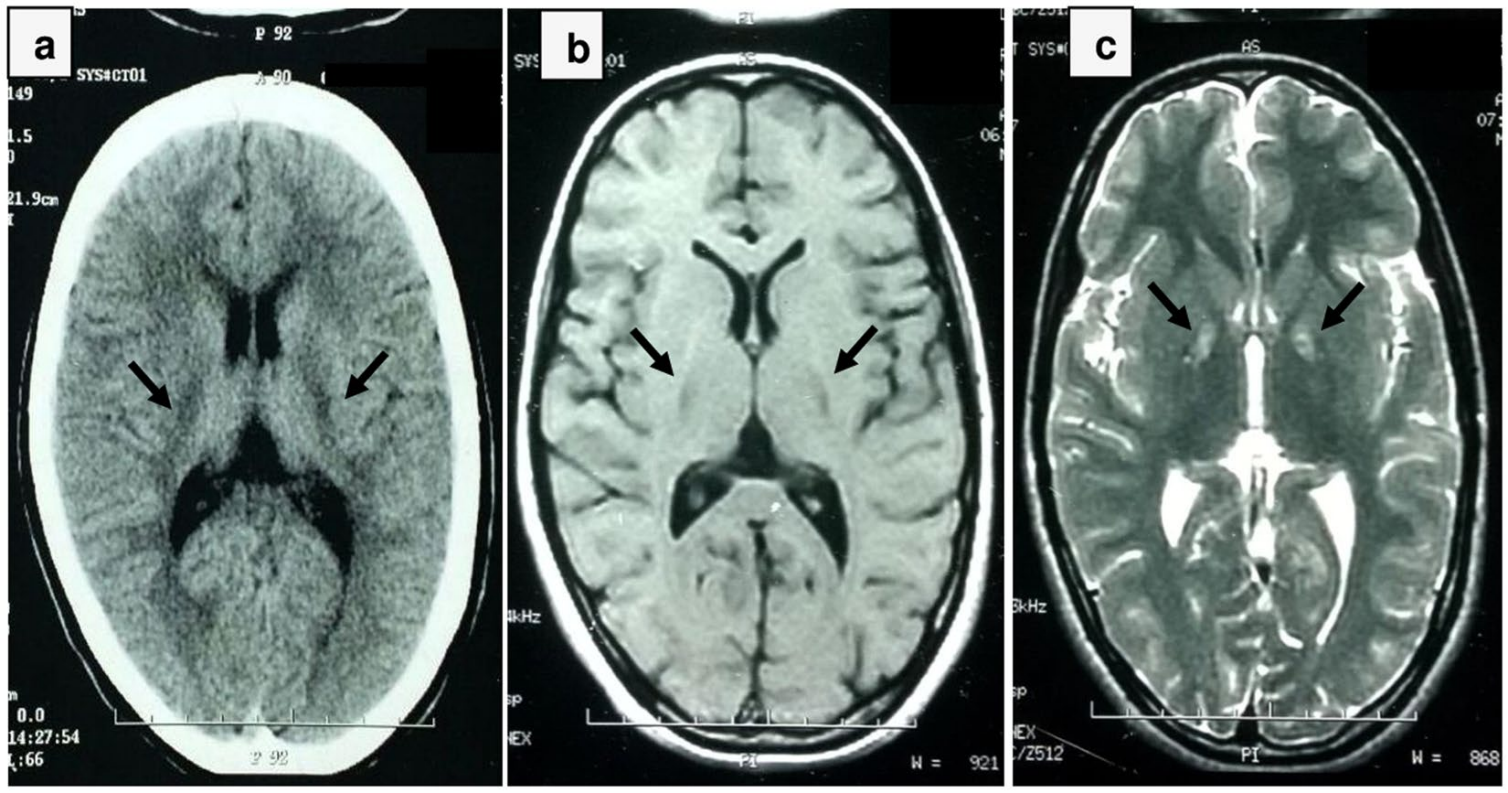
Brain MR-sequences of the proband of the study. Black arrows on the brain MR-sequences of the proband reveal (**a**) regions of hypointensities in bilateral globus pallidus in brain CT-sequence, (**b**) regions of hypointensities in bilateral globus pallidus on brain 0.2 T T1W MR-sequence, and (**c**) regions of hyperintensities in bilateral globus pallidus on brain 0.2 T T2W MR-sequence.

The proband, a 25 years old female, presented with an extra-pyramidal movement disorder. On examination, she displayed extra-pyramidal symptoms such as primary dystonia, spastic unsteady gait and mild dysarthria, and difficulty in walking. She had a dystonic foot and always needed a support to walk. She also complained of pain in her right upper limb and also displayed Babinski sign (upgoing plantars). However, her cognitive abilities were relatively preserved, and she was cooperative during examination. Her sensations were intact and superficial reflexes were easily elicitable. Other deep tendon reflexes were brisk. The power was grade IV in both upper and lower limbs. Her history revealed a delay in her developmental milestones and started walking at the age of 2. She faced difficulty in walking from her early childhood, accompanied by a tendency of frequent falling and gait dysfunction. She developed a valgus deformity with dystonia at the age of 4. She also developed dysphagia and she was unable to speak multisyllable words glibly. By her thirteenth year, symptoms started becoming progressive. Her speech became progressively slurred. Extensive laboratory workup showed normal blood histological profile without any acanthocytes and unremarkable results for liver and renal function tests, and plasma ceruloplasmin, lactate and copper urinary levels. Audiometric tests revealed a mild bilateral conduction hearing loss. There was no evidence of retinitis pigmentosa. T2W/FLAIR MRI investigations, conducted on the available Signa Profile 0.2 T MRI setup (GE Healthcare, Little Chalfont, United Kingdom), depicted a bilaterally symmetrical region of hyper-intensity in both globus pallidi, along with an area of hypo-intense signal on T1W sequences, whereas CT images reveal regions of hypo-intensity in bilateral globus pallidus (Fig. 1). The normal grey matter-white matter interface was found to be well-preserved on all MR-sequences.

Like his affected sister, the affected male also presented with a similar extra-pyramidal movement disorder as well as multiple episodic seizures. On examination, he was diagnosed with a dystonic foot, progressive mouth-tongue dystonia, and facial and perioral dyskinesia. He had a spastic gait and faced difficulty in walking. He showed decreased responsiveness to verbal commands, and a habit of irrelevant talking. He had an impaired memory with failure in identifying his own family members. His superficial and deep tendon reflexes were elicitable. However, his higher motor functions and power could not be assessed as the patient was not cooperative. His developmental history was not significant. By his thirteenth year, symptoms became progressive, characterized by frequent seizures and progressive impairment of cognition and speech. Extensive laboratory workup showed normal blood histological profile and unremarkable results for liver and renal function tests, and plasma ceruloplasmin, lactate and copper urinary levels. T2W/FLAIR MR-investigation depicts a region of hyper-intensity with subtle peripheral hypo-intensity in the lateral part of bilateral globus pallidi on T2W coronal sequence, along with an area of hypo-intense signal within the hyper-intense focal calcification region of both globus pallidi on all sequences (axial, coronal, sagittal, DW1 and DW2). No abnormal mass lesion was observed in the brain. The corpus callosum appeared normal, and also the normal grey matter-white matter differentiation was maintained.

## Methods

### Recruitment of the subjects

**Figure 2:**
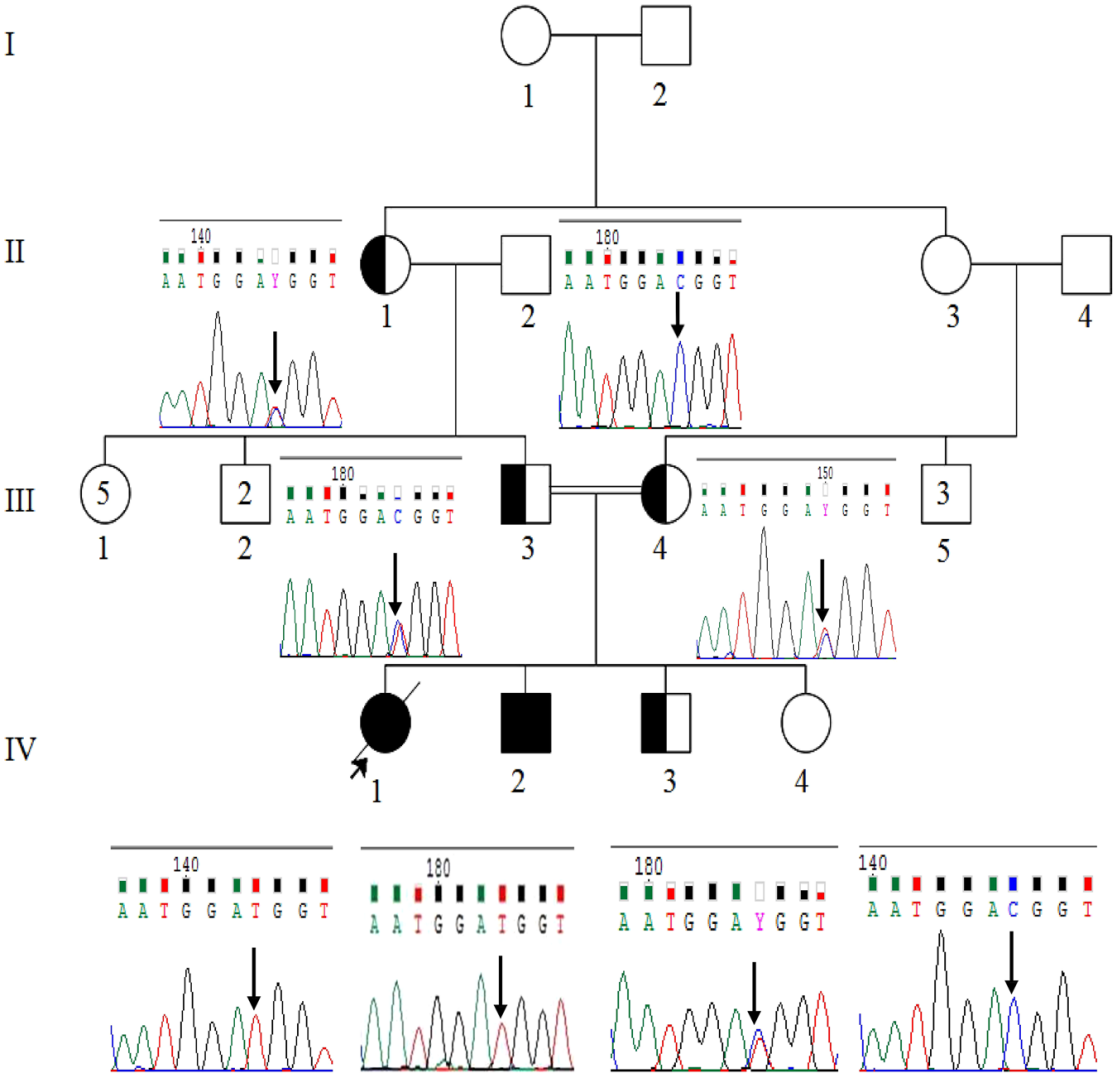
Pedigree of the PKAN family along with their representative sequence electrophoregrams indicating the autosomal recessive mode of transmission of NM_153638.3: c. 1069C > T *PANK2* variation. The proband IV(1) and her affected sibling IV(2) were found to be homozygous (TT) for the variation, and II(1), III(3), III(4) and IV(3) to be carriers (CT) of the variation whereas this variation was found to be absent in II(2) and IV(3). *Squares* represent the males and *circles* females, *filled symbols* affected, *unfilled* unaffected, *partially-filled* carriers and *arrows* mark the proband.

After their clinical and radiological examination at a local hospital, both patients and their unaffected family members including their paternal grandparents, parents and siblings (Fig. 2) were recruited for genetic screening of the *PANK2* gene. All the subjects were included in the study after obtaining their/their parents’ informed consent for participation according to the Declaration of Helsinki. Unfortunately, the proband of this study had deceased recently. The study was approved by the Institutional Ethical Review Board (IERB) committee at Shri Mata Vaishno Devi University, Katra, J&K.

### Isolation of DNA from blood and saliva samples

About 2 ml of whole peripheral blood sample from the patients, their parents and two normal siblings through venipuncture and about 2 ml of saliva samples from their paternal grandparents were procured. Genomic DNA was extracted from peripheral white blood cells using FlexiGene® DNA kit, QIAGEN, Cat No. 51206 and from saliva using DNeasy® Blood & Tissue kit, QIAGEN, Cat No. 69504.

### Genetic evaluation: Direct Sanger sequencing of *PANK2* gene

Polymerase chain reaction (PCR) was performed to amplify *PANK2* exons 1 to 7 as well as their exon/intron boundaries using Mastercycler® pro, Eppendorf AG, Hamburg, Germany. We used the mRNARefSeq NM_153638.3 as a reference sequence for designing our primers. All the primer sequences used in this study are available on request. The PCR amplicons were purified using illustra^TM^ GFX^TM^ PCR DNA and Gel Band Purification Kit, Cat. No. 28–9034–70, GE Healthcare. For genetic evaluation, purified PCR amplicons were sequenced in both forward and reverse directions using dye terminator chemistry (BigDye® Terminator v3.1 Cycle Sequencing Kit) on an automated capillary sequencer (ABI 3500 Genetic Analyser, Applied Biosystems®, Foster City, California, USA). Sequence electrophoregrams were analyzed using the Sequence Scanner Software v2.0 (Applied Biosystems®) and Chromas v2.5.1 (Technelysium Pty Ltd, South Brisbane, Australia). Lasergene Genomics Suite of DNASTAR® software, DNASTAR Inc., USA was used for sequence alignments and for evaluation of the genetic variations.

### *In-silico* analyses

The Mutation Taster^24^ (http://mutationtaster.org/), PolyPhen-2^25^ (http://genetics.bwh.harvard.edu/pph2/) and SIFT^26^ (http://sift.jcvi.org/) were used to evaluate potential pathogenic nature of the observed *PANK2* variant. Many other online databases like BLAT, PDB and tools like PyMOL were used. For conducting molecular dynamics studies, Protein Preparation Wizard of Schrodinger Maestro provided along with the Academic Version of Desmond MD simulation tool v35014 was used. Root Mean Square Deviation (RMSD), Radius of Gyration and angle measurements were done through Simulation Event Analysis module of Schrodinger Maestro. Root Mean Square Fluctuation (RMSF) and Principle Component Analysis (PCA) was done through the Bio3D^27^(an R based package).

## Results

### Clinical diagnosis of the disorder

The patients were diagnosed with PKAN on the basis of their clinical and radiological evaluation.

### Genetic evaluation: Sequencing of *PANK2* gene revealed a missense variation in its exon 3

Sanger sequencing of all the PCR-amplified exons of *PANK2* gene revealed a familial, missense variation NM_153638.3: c. 1069C > T (rs753376100) in its exon 3, segregating in the family in an autosomal recessive mode. Both patients were found to be homozygous for the variation. Both of their parents, paternal grandmother and healthy brother were found to be asymptomatic carriers of the variant allele, whereas their paternal grandfather and healthy sister were found to be homozygous wild. The electrophoregrams indicating nucleotide position of the observed *PANK2* exon3 (rs753376100) variant are represented along with the pedigree in Fig. 2. *In-silico* analyses through Mutation Taster, PolyPhen-2 and SIFT predicts pathogenic nature of this variation with a high probability (Mutation Taster p-score = 0.99989, PolyPhen-2 p-score = 0.996 and SIFT score = 0.03, predicted as damaging).

### *In-silico* analyses of the c. 1069C > T variation

**Figure 3:**
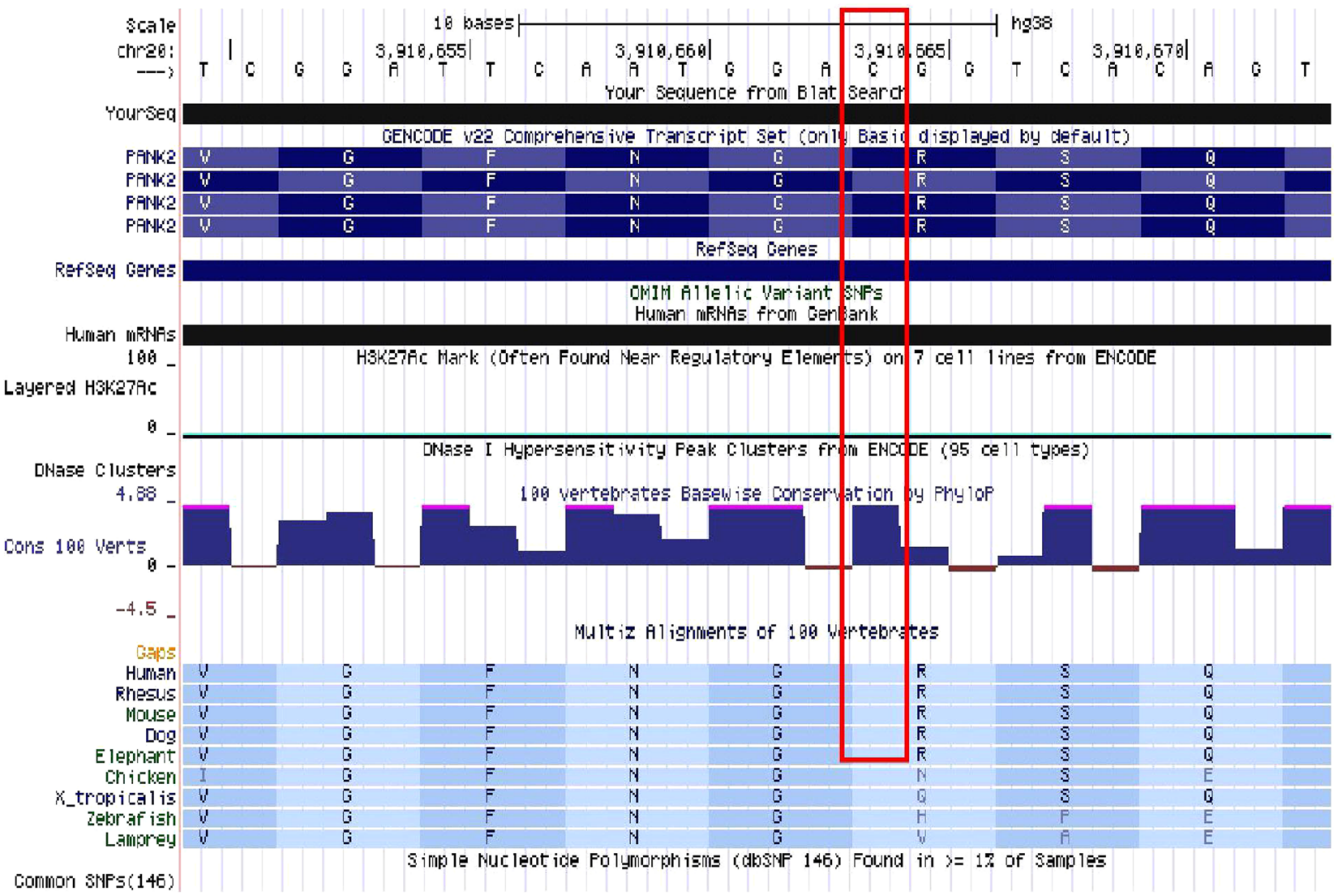
Screenshot from UCSC Genome Browser representing conservation of the variant nucleotide (NM_153638.3: c. 1069C > T) of the *PANK2* exon 3 across 100 vertebrates.

**Figure 4:**
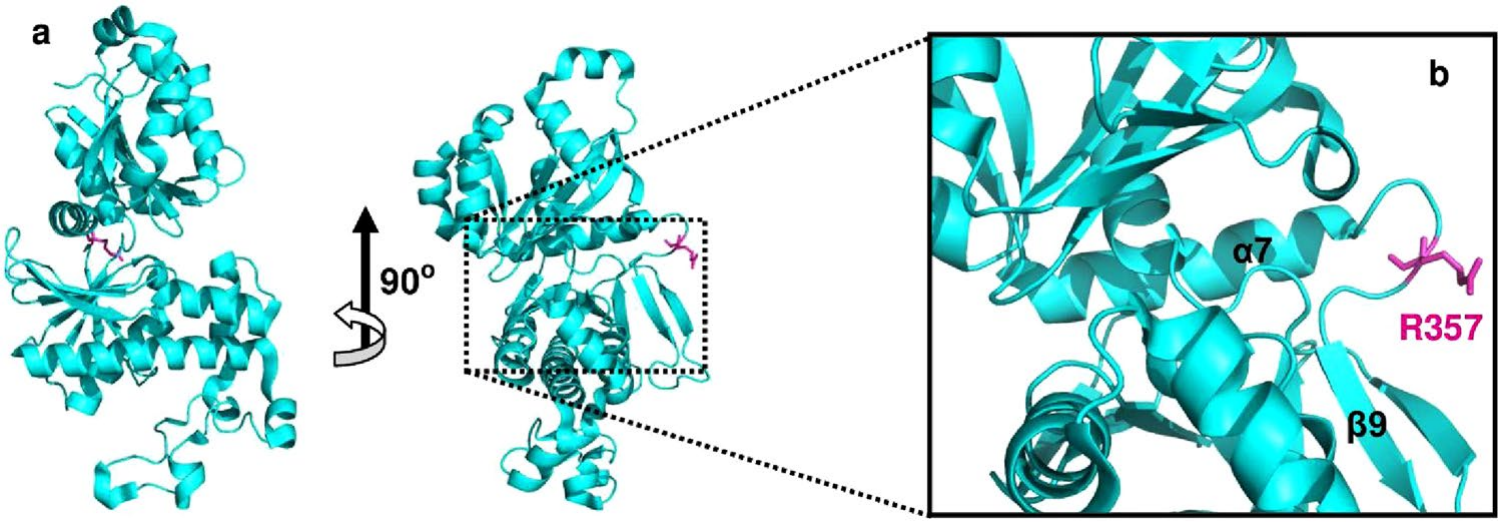
Mapping of the PKAN-associated R357W variation on the hPanK2 structure (5e26.pdb). (**a**) Blue cartoon representation of a wild-type hPanK2 monomer indicating the position of wild R357 (shown as light magenta). (**b**) The magnified image reveals that the R357 lies onto the A-domain of the PanK2 monomer.

The c.1069C > T missense variation in the *PANK2* gene was found to be present at the first highly conserved nucleotide base position of codon 357 throughout most of the mammalian species(Fig. 3). This variation resulted in an amino acid change from polar, positively charged Arginine (R) to non-polar, neutral Tryptophan (W) at position 357 (p. (Arg357Trp)) of the hPanK2 protein. Crystal structure of hPanK2 was retrieved from PDB (5e26.pdb). The R357W variation was found to be a surface variation and can be mapped to a β-bridge within the A-domain of hPanK2 by using the available structures of the highly homologous PanK1 (PDB: 2I7N) and PanK3 (PDB: 2I7P) proteins as models (Fig. 4(B)). Since thec.1069C > T variation was identified at a highly conserved region of the *PANK2* codon 357 and resulted in an amino acid change (W)entirely different in nature from the wild-type amino acid (R), it was considered that the resulted amino acid change may have some repercussions on the structure as well as functional dynamics of the hPanK2 protein.

In order to study and compare the effect of the observed c. 1069C > T variation on the overall dynamics of hPanK2 protein, four molecular dynamics simulations for wild-type Arg357 (R357), wild-type like Lys357 (K357; amino acid having similar chemical nature as Arginine), already known variant Gln357 (Q357; rs754521581) and our Trp357 (W357) variant were conducted for a time-lag of 100 nanoseconds. Single chain of hPanK2 was selected and refined by the addition of the missing side chains online at http://lorentz.immstr.pasteur.fr. hPanK2 variants, that is, K357, Q357 and W357, were generated from the wild type hPanK2. Structures were further refined by addition of hydrogens, conversion of selenomethionines to methionines, capping of the terminal amino acids of the hPanK2 using the Protein Preparation Wizard of Schrodinger Maestro provided along with the Academic Version of Desmond MD simulation tool v35014. Water molecules and hetero-atoms were stripped off. The pKa values at physiological pH 7 were assigned by PROPKA tool. The resulting structures were then optimized and overlapping atoms were refined manually. For molecular dynamics simulations, system was build using the TIP3P solvation model in orthorhombic box shape. An OPLS 2005 force field was employed, and system was neutralized by the addition of Sodium and Chloride ions with an overall concentration of 0.15 molar. The NPT ensemble was employed, temperature was set to 300 Kelvin and pressure was set to 1 Bar. Smallest iteration step was 2 femtoseconds using RESPA integrator. Rest parameters for running the simulation were same as default. Analysis of results obtained from the molecular dynamics simulations revealed that the extended loop of hPanK2 has more mobility in case of R357 and variant K357 in comparison to the other hPanK2 variantsQ357 and W357. Plots of radius of gyration (see Supplementary Fig. 1b) indicated that the disease associated Q357 and W357 variants displayed more compactness than the wild-type PanK2and wild-type like variantas discussed above. Measure of the angle between the C-α of Isoleucine (I) 453 of extended loop, Leucine (L) 489 and Cysteine (C) 508 of helix 9 of B-domain of the hPanK2 shows that the extended loop of the W357 variant remained attached to its catalytic domain in comparison to the wild-type and other hPanK2 variants studied above (see Supplementary Fig. 1c and Supplementary Videos).Also, results of the RMSF analysis conducted for analysis of the fluctuation profile of the individual amino acid residues from the molecular dynamics simulation trajectories indicate that the peptide region (~20 residues) following the W357 residue forming the β-hairpin in W357 PanK2 variant (see Supplementary Fig. 3c) underwent greater fluctuation in comparison to R357, K357 and Q357 PanK2 (see Supplementary Fig. 3a,b and d, respectively, online), whereas region of the extended loop displayed comparatively lesser fluctuation than that of other PanK2 monomers R357, K357 and Q357. This partially supports our claim for the predicted rigid nature of the extended loop in case of the W357 PanK2 variant. We also suggest that the β-hairpin following the W357 residue could translate the allosteric signal required for the rigidity of the extended loop in W357 PanK2 variant. Also, through the PCA, it has been found out that in W357 variant the amino acid residues 358–377 of the β-hairpin following W357 contributed greater to the first three Principal Components (PCs) (see Supplementary Fig. 4c), whereas these residues in R357 and K357 PanK2 (see Supplementary Fig. 4a and b) reported comparatively lesser contribution to the first three PCs. In addition the residues comprising the extended loop in R357 and K357 sampled through greater conformational space as evident from their greater contribution to PC1, PC2 and PC3 compared to Q357 and W357.

## Discussion

The PKAN is a rare hereditary neurodegenerative disorder associated with aberrant non-heme iron accumulation in the neurons of the globus pallidus and substantia nigra regions of the basal ganglia. In this case report, we present a familial case of classic PKAN from J&K, India, for the first time, associated with c.1069C > T missense variation (rs753376100) in exon 3 of *PANK2* gene. The clinical phenotype of our patients was found to be concordant with classic PKAN in terms of the age of onset, symptoms and radiological findings, as described earlier. The patients were found to be homozygous for c.1069C > T variation. Also, this variation had segregated in the patients’ family in an autosomal recessive mode. This *PANK2* variation has led to a surface amino acid residue change p.(Arg357Trp), the variant amino acid residue was mapped to a β-bridge within the protein’s catalytic A-domain by using homologous PanK1 and PanK3 as protein models.

Although several functional studies have been conducted for understanding the pathophysiology of PKAN, but a detailed insight into its underlying molecular pathogenesis is yet to be delineated. A growing number of experimental evidences suggest that the characteristic phenotype of the disease, that is, aberrant iron accumulation in the brain, is a secondary phenomenon in its pathogenesis associated with disruption in the CoA homeostasis due to defective PanK2 protein. It has been proposed that the loss of PanK2 function and deficiency of phosphopantothenate due to *PANK2* nucleotide variations are associated with deficiency of mitochondrial CoA and reduction in the activity of the enzyme cysteine dioxygenase (EC 1.13.11.20) in highly metabolically active organs, such as the globus pallidus and retina which subsequently results in localized accumulation of cysteine-rich substrates (CRS) that in conjunction with iron either directly or indirectly cause increased oxidative damage in the more susceptible areas of the brain^23^. However, PKAN-associated *PANK2* variations are known to alter the function of hPanK2 by disrupting the process of its maturation and mitochondrial localization as well as its catalytic activity, whereas *in vitro* studies for some of these nucleotide variations have not revealed any disruption in the hPanK2 catalytic activity, suggesting additional mechanisms through which hPanK2 function can be disrupted in mitochondria due to*PANK2* variations^23^. These additional mechanisms remain to be elucidated.

The PanK proteins are known to function as homodimers; the dimerization interface being constructed between the β-strands of B-domain of one PanK monomer and the extended loop of the other monomer (a head-to-tail juxtaposition), contributing to the stabilization of the PanK dimer^28, 29^. Some of the PanK2 missense variations associated with both the classic and atypical form of PKAN have been mapped to the active site, dimerization interface; the protein core and the surface residues of a highly homologous PanK3 protein and have been implicated for the inactivation and destabilization of PanK3 as well as reduction in its activity^28^. However, the results of our *in-silico* study for the PanK2 variants Q357 and W357 have indicated that the extended loop of the kinase monomers remained associated with their respective catalytic domain, as found similar to the crystallized homo-dimerized form of PanK2 (PDB ID: 5E26). Based on this observation, we suggest that R357 residue could be essential for the stability of the catalytic domain of the PanK2 and p.(Arg357Trp) variation may have a structural as well as functional implication on PanK2 by disrupting dimerization of the PanK2 monomers and compromising its catalytic activity *in vivo*.

The catalytic activity of mammalian PanK isoforms is particularly regulated by acetyl-CoA (IC_50_ = 0.1 µM) through a mechanism of feedback inhibition^30, 31^. It has been observed that acetyl CoA binds at an allosteric site (a linear pocket near the catalytic site) spanning the dimer interface of PanKs and renders the substrate-binding site in an inactive open conformation which is unfavourable for the binding of ATP at the catalytic site, thus inhibiting the kinase reaction^28, 29, 32^. The α, phosphates of acetyl CoA and β, γ phosphates of the ATP share common binding surface on PanKs. We superimposed the crystal structure of PanK2 with Pank1 and PanK3, and found that amino acid residues near the Acetyl CoA were both sequentially and spatially conserved across human PANKs (see Supplementary Fig. 2b). We also observed that acetyl CoA binds PanK2 at the dimerization interface formed between the β-strands of B-domain of one monomer and the extended loop of the other monomer (see Supplementary Fig. 2a). These observations may add another insight into the dynamic behavior of the PanK2 protein. We suggest that the dynamic nature of the extended loop could be responsible for the active dimerized conformation of the PanK2 protein and its rigidity in case of the disease-associated PanK2 variant may stabilize the acetyl CoA-bound dimerized inactive conformation of the PanK2. This could partially be supported by the results of other molecular dynamics analysis like RMSF and PCA.

In conclusion, the patients presented herein were diagnosed with early-onset classic type of PKAN according to their clinical presentation, age-of-onset of symptoms and genetic evaluation of an already known disease-associated *PANK2* gene. We found a c. 1069C > T missense variation (rs753376100) in exon 3 of the *PANK2* gene that segregated in the family in an autosomal recessive manner and depicted a surface amino acid change p.(Arg357Trp). However, a p.(Arg357Gln) (rs754521581) variation at the same amino acid position is already known. Our comparative molecular dynamics study for the monomers of wild-type PanK2 (R357) and three PanK2variants(K357, W357 and Q357) indicated a rigid nature of the extended loop in case of our disease-associated PanK2 variantW357 as well as in other already known PanK2 variant Q357. We suggest that R357 residue could be essential for the stability of the catalytic domain of the PanK2. Missense variations at this position may have a structural as well as functional implication on PanK2 by interfering with the dimerization of the PanK2 monomers, resulting in non-dimerized and inactive PanK2 with a compromised catalytic activity *in vivo*. Also, the comparative rigidity of the extended loop in case of the disease associated variant may be stabilizing the Acetyl CoA bound dimerized inactive conformational state of PanK2, and render structure of the W357 PanK2 variant unfavourable for carrying out its kinase activity. The biochemical and biophysical characterization of the W357 variant with respect to dimerization, protein stability and its catalytic activity may serve as a possible model for understanding the implication of PanK2 variations to PKAN pathogenesis. This is, however, beyond the scope of the current study.

## Electronic supplementary material

Molecular dynamics of wild type hPanK2 having R357

Molecular dynamics of wild type like hPanK2 having K357

Molecular dynamics of mutant hPanK2 having W357

Molecular dynamics of already known mutant hPanK2 having Q357

Supplementary Figures 1-5
