# Supplementary material for: A variation in PANK2 gene is causing Pantothenate kinase-associated Neurodegeneration in a family from Jammu and Kashmir – India
Source: Sci Rep. 2017 Jul 5;7:4834. doi: 10.1038/s41598-017-05388-9 (PMC5498598; doi:10.1038/s41598-017-05388-9)
Supplement: Supplementary file 5 — Supplementary Figures 1-5 [file 41598_2017_5388_MOESM5_ESM.pdf]

# A variation in *PANK2* gene is causing Pantothenate kinase-associated Neurodegeneration in a family from Jammu and Kashmir – India

**Arshia Angural[1], Inderpal Singh[2,+], Ankit Mahajan[3,+], Pranav Pandoh[4,+], Manoj K Dhar[3], Sanjana Kaul[3], Vijeshwar Verma[2], Ekta Rai[1], Sushil Razdan[5], Kamal Kishore Pandita[6*] & Swarkar Sharma[1,*]**

[1]Human Genetics Research Group, Department of Biotechnology, Shri Mata Vaishno Devi University, Katra, Jammu and Kashmir, India,182320.
[2]Bioinformatics Infrastructure Facility, Department of Biotechnology, Shri Mata Vaishno Devi University, Katra, Jammu and Kashmir, India, 182320.
[3]Department of Biotechnology, University of Jammu, Jammu and Kashmir, India, 180006.
[4]Acharya Shri Chander College of Medical Sciences and Hospital, Sidra, Jammu, Jammu and Kashmir, India, 180017.
[5]Bhagwati Nagar, Jammu, J&K, India.
[6]H. No. 62, Lane 11, Swam Vihar, Muthi, Jammu, J&K, India.

[+]These authors contributed equally to this work.
*Correspondence and request for materials should be addressed to K.K.P. (email: panditakk 69@gmail.com) or S.S. (email: swarkar.sharma@smvdu.ac.in)

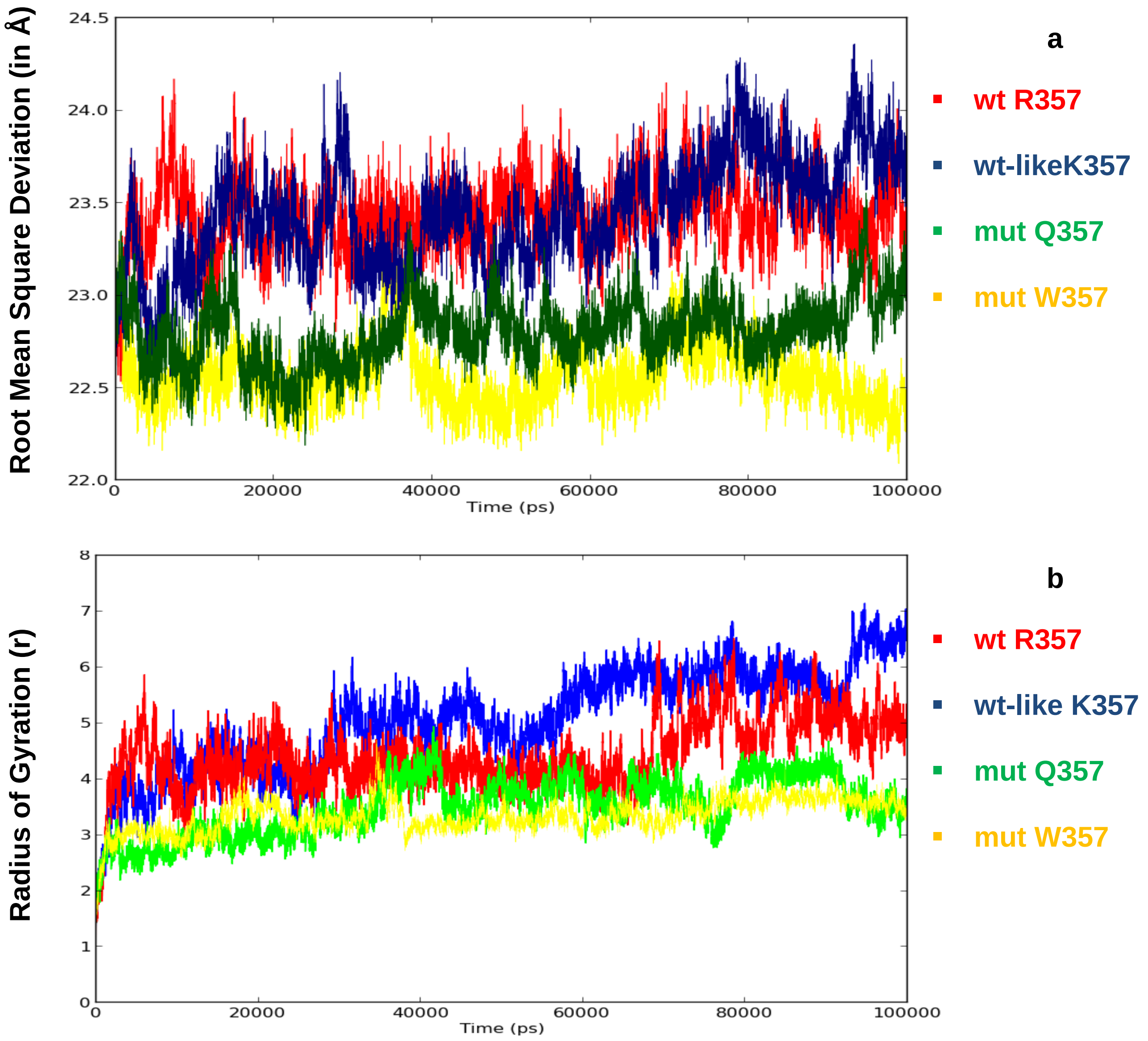

a
Root Mean Square Deviation (in Å)
24.5
24.0
23.5
23.0
22.5
22.0
0
20000
40000
60000
80000
100000
Time (ps)
wt R357
wt-likeK357
mut Q357
mut W357
b
Radius of Gyration (r)
8
7
6
5
4
3
2
1
0
0
20000
40000
60000
80000
100000
Time (ps)
wt R357
wt-like K357
mut Q357
mut W357

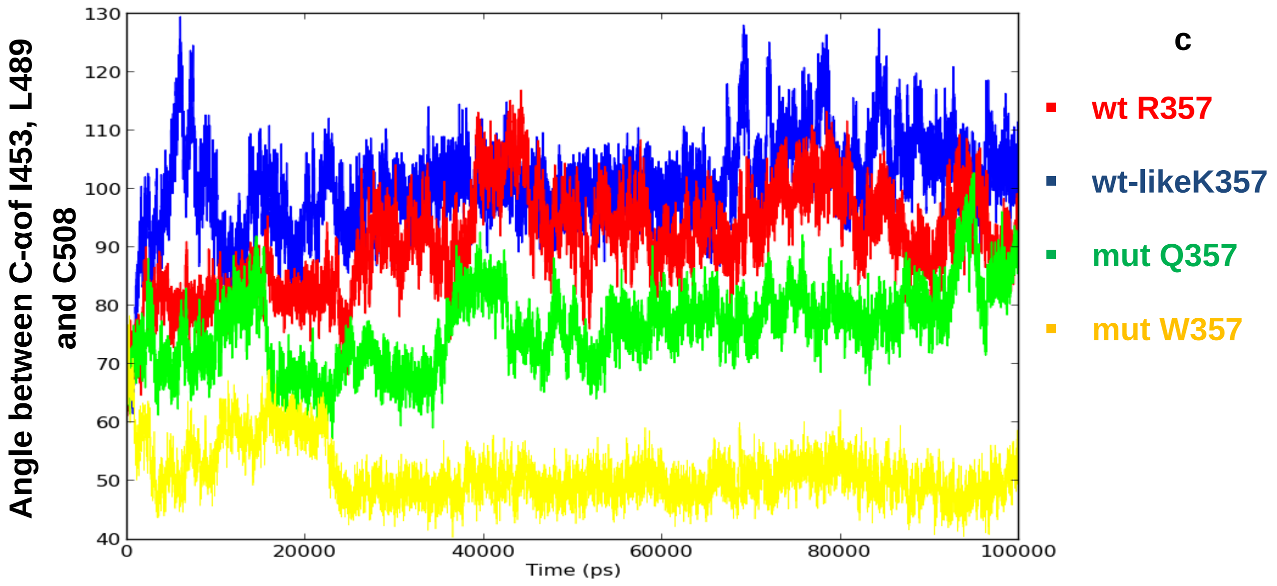


**Supplementary Figure 1. Results of the molecular dynamics for the wild-type (wt) PanK2 with Arginine (R) at 357 (R357) and three PanK2 variants: wild-type like Lysine (K) (K357), already known disease-associated Glutamine (Q) (Q357) and Tryptophan (W) (W357).** (a) In molecular dynamics simulations, RMSD is the measure of the average distance between the atoms (in this case non-Hydrogen atoms) of the superimposed protein conformations extracted from the molecular dynamics trajectory over equal intervals. Here, the plot shows that the disease associated variants (Q357 and W357) report less RMS deviation in comparison to the wt-R357 and wt-like K357 variant. (b) Radius of gyration is the measure of the compactness of protein during molecular dynamics simulation. The plot shows that the disease associated variants W357 and Q357 have less radius of gyration, and thus they were more compact compared to the R357 and K357 variant during 100 ns long simulation. (c) The plot for the angle between C-α of Isoleucine (I) 453 of extended loop and Leucine (L) 489 & Cysteine (C) 508 of helix 9 of B-domain of the hPanK2 shows that the extended loop of the W357 variant remained attached to its catalytic domain in comparison to the other forms of PanK2 studied above.

a

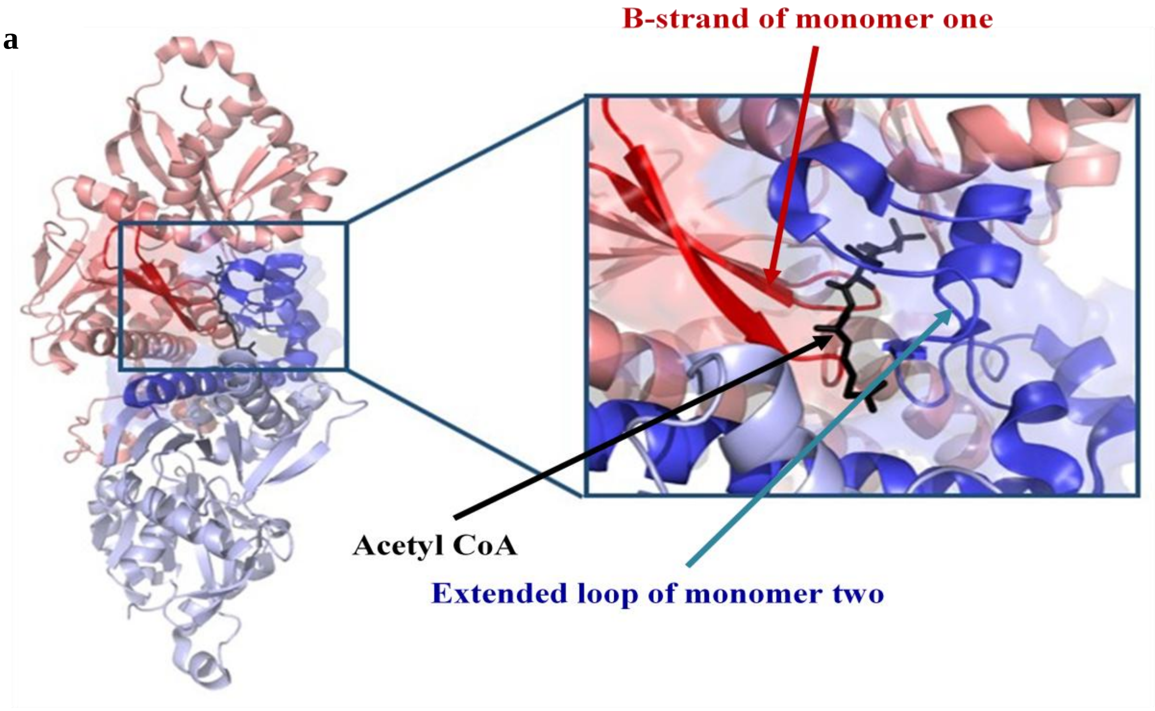


b

**Residues around the Acetyl CoA are conserved across hPANKs**

**Residues around the Acetyl CoA in and around the beta strands of monomer one**

---Ile$^{390}$ Gly$^{391}$ Ser$^{392}$ Gly$^{393}$ Val$^{394}$ Ser$^{395}$ --- ---Thr$^{409}$ Gly$^{410}$ Thr$^{411}$ Ser$^{412}$ --- (PANK2 5E26.PDB)

---Met$^{415}$ Gly$^{416}$ Ser$^{417}$ Gly$^{418}$ Val$^{419}$ Ser$^{429}$ --- ---Thr$^{434}$ Gly$^{435}$ Thr$^{436}$ Ser$^{437}$--- (PANK1 2I7N.PDB)

---Ile$^{190}$ Gly$^{191}$ Ser$^{192}$ Gly$^{193}$ Val$^{194}$ Ser$^{195}$ --- ---Thr$^{209}$ Gly$^{210}$ Thr$^{211}$ Ser$^{212}$--- (PANK3 2I7P.PDB)

**Residues around the Acetyl CoA in and around the extended loop of monomer two**

--Val$^{450}$-- --Ala$^{467}$ Val$^{468}$ Ala$^{469}$ Ser$^{470}$ -- --Asn$^{499}$-- --Ala$^{537}$-- --Tyr$^{540}$-- (PANK2 5E26.PDB)

--Val$^{475}$-- --Ala$^{492}$ Val$^{493}$ Ala$^{494}$ Ser$^{495}$ -- --Asn$^{524}$-- --Ala$^{562}$-- --Phe$^{565}$-- (PANK1 2I7N.PDB)

--Val$^{250}$-- --Ala$^{267}$ Val$^{268}$ Ala$^{269}$ Ser$^{270}$ -- --Asn$^{299}$-- --Ala$^{337}$-- --Phe$^{340}$-- (PANK3 2I7P.PDB)

**Supplementary Figure 2. Mapping of the Acetyl CoA-binding sites in the hPanK2.** (a) Cartoon representation of dimerised hPANK in complex with Acetyl CoA highlighting the secondary structure elements, beta strand of monomer one (colored in dark red ) and extended loop of monomer two (colored in dark blue), involved in the binding to Acetyl CoA (colored in black). (b) Representation of sequentially and spatially conserved amino acids around the bound Acetyl CoA across hPANK1, hPANK2 & hPANK3.

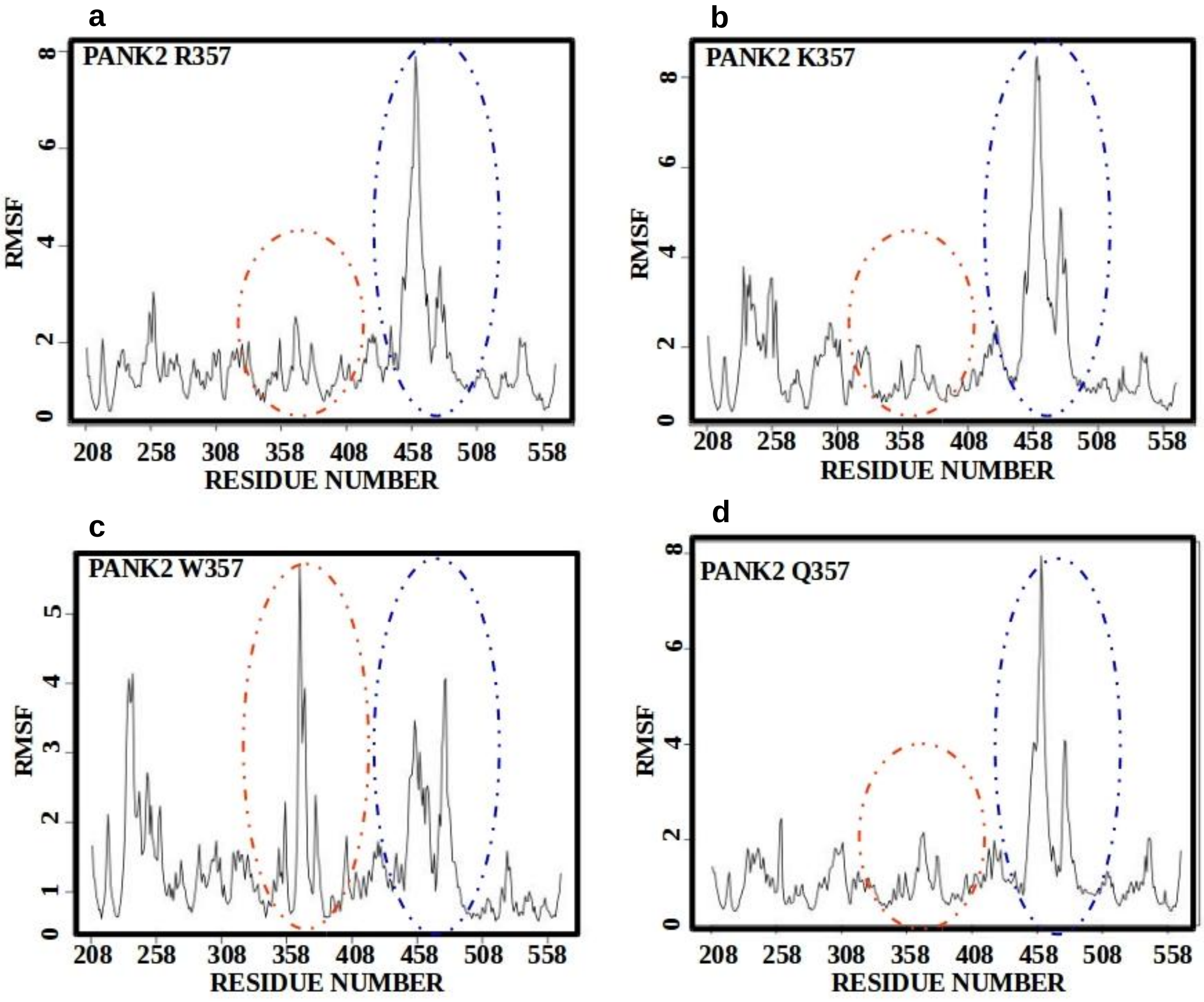


**Supplementary Figure 3. The plots for the RMSF analysis indicating the fluctuation profile of the individual PanK2 amino acid residues.** To calculate the fluctuation profile of the individual amino acids from the MD simulation trajectories, we performed the RMSF analysis. The red circles on the RMSF plots indicate that the peptide region (~20 residues) following W357 residue forming the β-hairpin in W357 PanK2 variant (c) underwent greater fluctuation compared to R357 (a), K357 (b) and Q357 PanK2 (d). Region of the extended loop indicated through blue circles in case of W357 PanK2 reported comparatively lesser fluctuation than the R357, K357 and Q357 PanK2. We conclude that the β-hairpin following the W357 residue translated the allosteric signal required for the rigidity of the extended loop in W357 PanK2 variant.

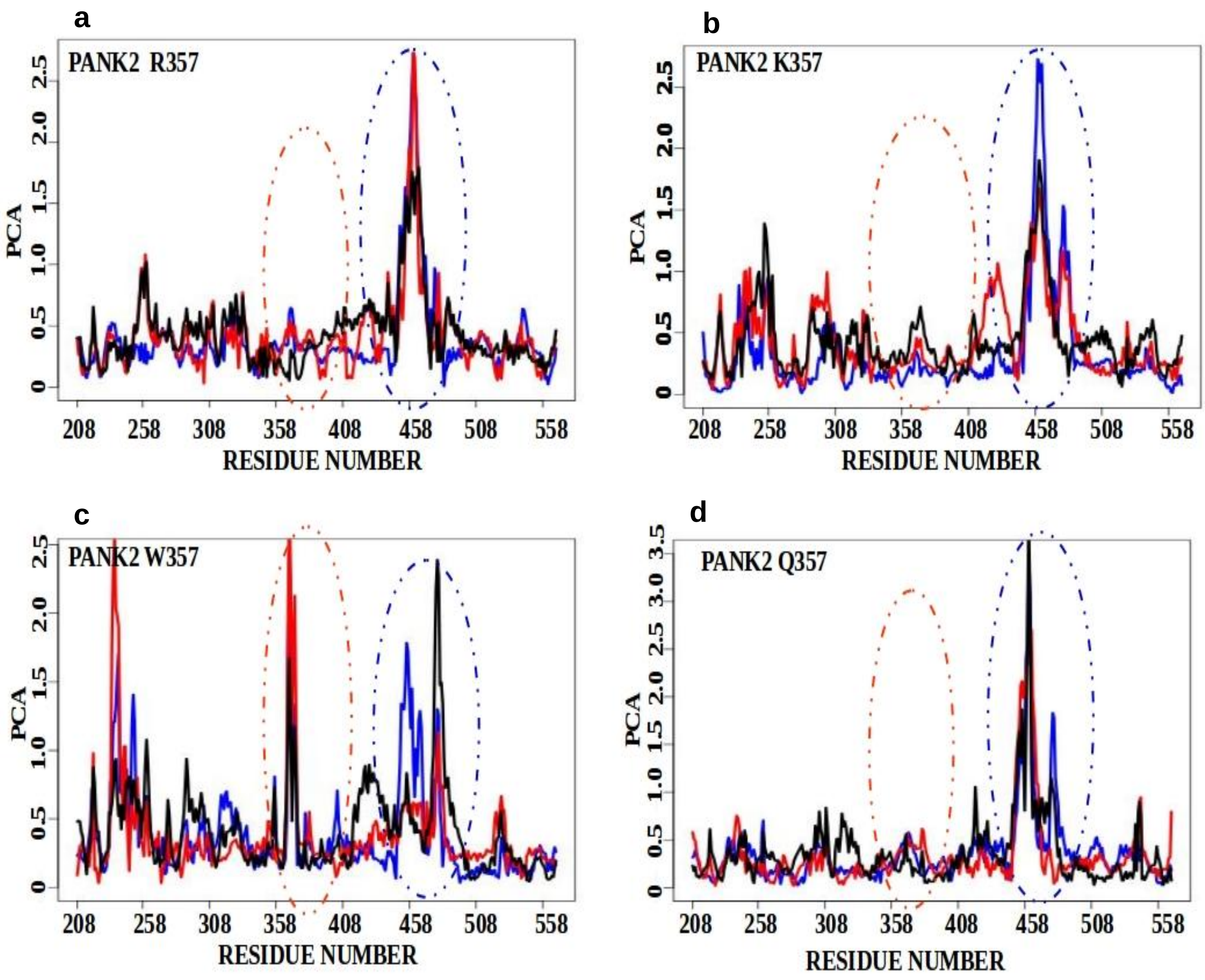


**Supplementary Figure 4. The plots for per residue contribution to first three Principal Components (PCs) for R357, K357, W357 and Q357 PanK2.** PCA analysis is done for determining the essential dynamics of protein during the molecular dynamics simulation. Here, the per-residue contribution to the first three PCs is plotted (PC1 - blue; PC2 - red; PC3 - black). Amino acid residues belonging to the extended loop reported greater contribution to the first two PCs and thus sampled through greater conformational space in R357 (a) and K357 (b) PanK2s than Q357 (d) and W357 (c) PanK2 variants, whereas 357-377 residues in W357 variant contributed greater to the first three PCs than the equivalent residues in the PanK2 R357, K357 and Q357.

**αC PCA of PANK2 R357**

**a**

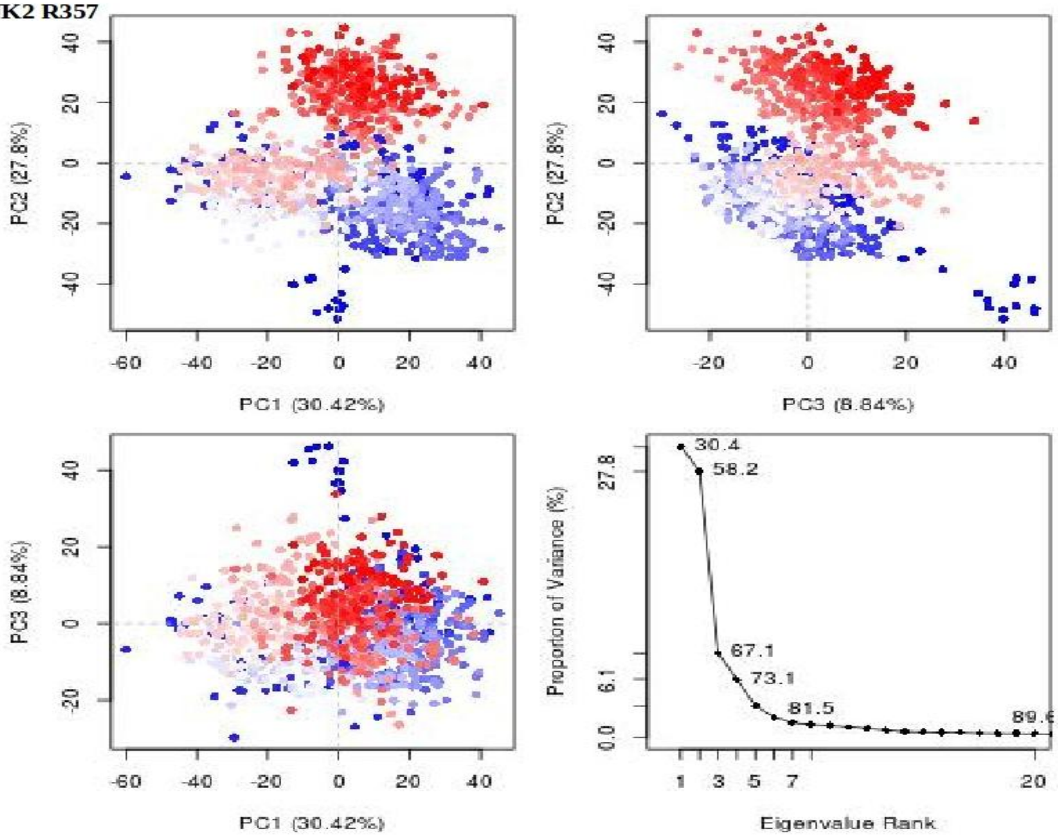


**αC PCA of PANK2 K357**

**b**

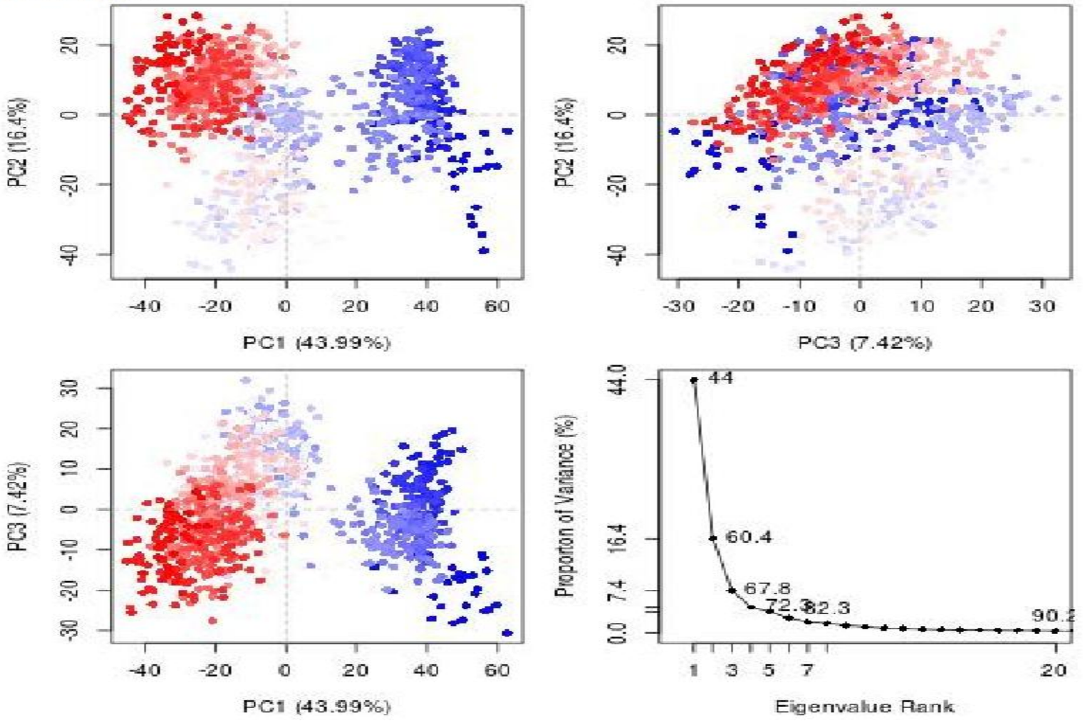

**αC PCA of PANK2 W 357**

c

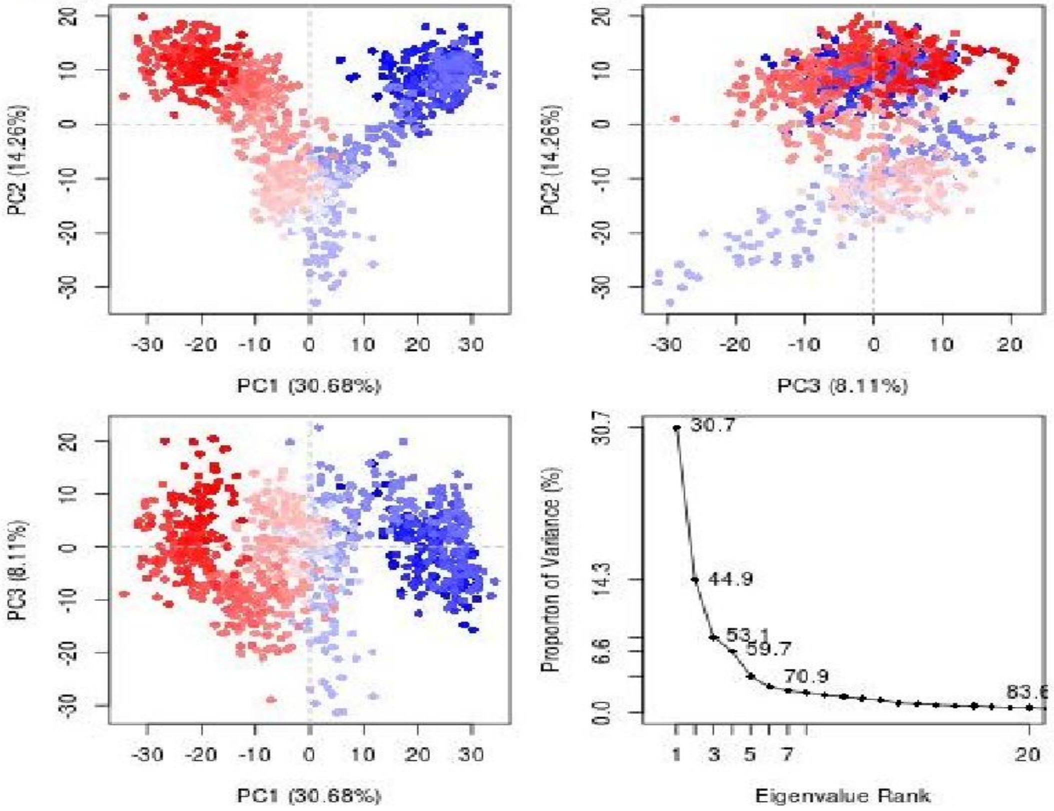


**αC PCA of PANK2 Q357**

d

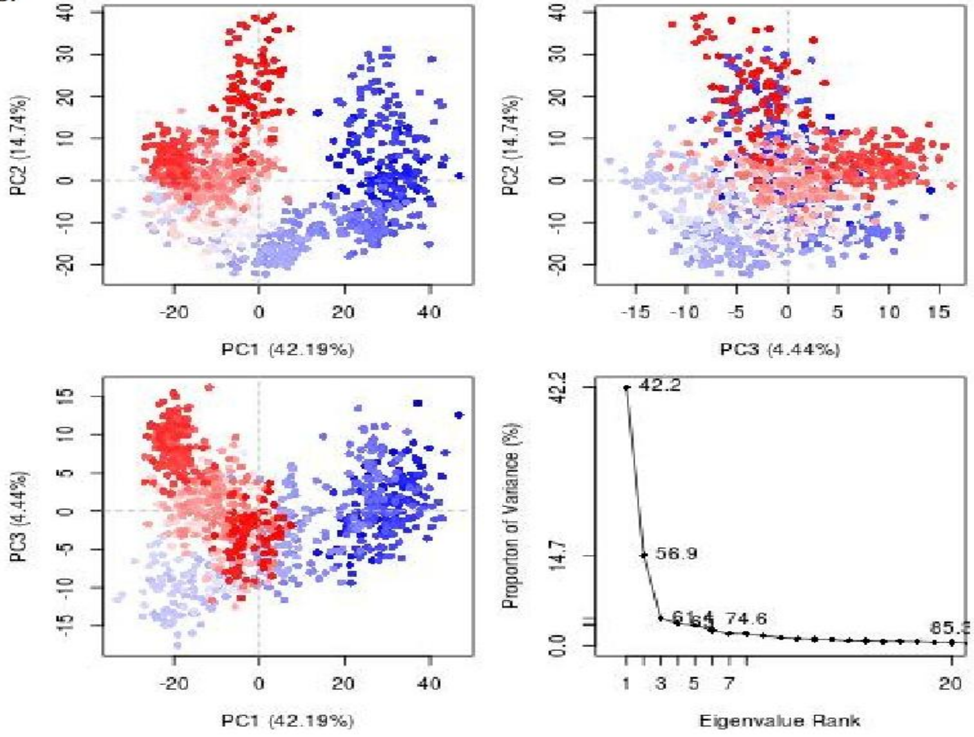

**Supplementary Figure 5. PCA conformer plot for first three PCs including the skew plot indicating the proportion of variance for Cα-PCA of R357 (a), K357 (b), W357 (c), and Q357 (d).** PCA plots for the instantaneous 1000 trajectory frames taken at equal intervals from the 100 ns simulation are colored from blue to red in the order of time. Also, included are the skew plots of proportion of variance for R357, K357, W357 and Q357. For R357, the first three PCs collectively account for 67.1% mean square deviation in the original coordinate data, whereas for K357, W357 and Q357 the first three PCs collectively account for 67.8%, 53.1% and 61.4%, respectively.
